# Supplementary material for: Drug repurposing for aging research using model organisms
Source: Aging Cell. 2017 Jun 16;16(5):1006–15. doi: 10.1111/acel.12626 (PMC5595691; doi:10.1111/acel.12626)
Supplement: Supplementary file 7 — Data S1 Zip‐Archive of all report cards. [file ACEL-16-1006-s007.zip › RC_LPR.pdf]

LPR

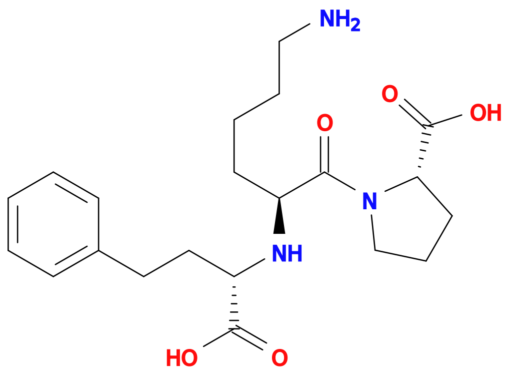

Database identifiers

ChEMBLCompound	CHEMBL1237
DrugBank	DB00722
CHEBI	43755
ZINC	ZINC03812863
eMolecules	4775693

Ranking

	Rank	Score
Drosophila	297/697	0.558
C. elegans	233/591	0.232

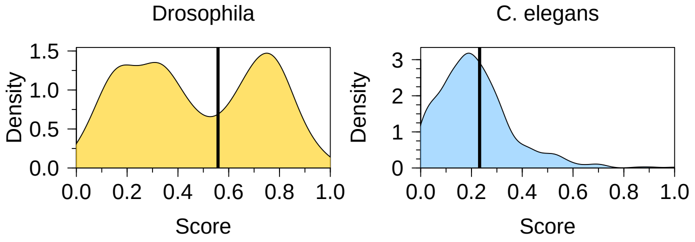

	Ageing implication	Domain conservation	Binding site conservation	Binding affinity	Bioavailability	Lipinski	Promiscuity	Purchasability	Drug approval	**Total**
Drosophila	0.476	0.982	0.927	0.918	(0.9)	0.0	-0.0	0.1	0.1	0.558
C. elegans	0.476	0.746	0.48	0.918	0.208	0.0	-0.0	0.1	0.1	0.232

Names

- **lisinopril**
- (S)-1-(N(2)-(1-carboxy-3-phenylpropyl)-L-lysyl)-L-proline
- n2-(s)-1-carboxy-3-phenylpropyl-l-lysyl-l-proline
- Prinivil
- Zestril

Roles

EC 3.4.15.1 (peptidyl-dipeptidase A) inhibitor

Status

Approved drug (according to ChEMBL)	Yes
Administration Route	Oral
Number of Rule of 5 violations	0
Binding affinity to original target in log units (RF-Score prediction)	7.42
Burns *C. elegans* bioavailability prediction	-6.41

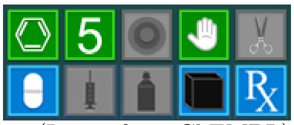
(Image from ChEMBL)

Medical Information

Indicatation: For the treatment of hypertension and symptomatic congestive heart failure. May be used in conjunction with thrombolytic agents, aspirin and/or amp;beta;-blockers to improve survival in hemodynamically stable individuals following myocardial infarction. May be used to slow the progression of renal disease in hypertensive patients with diabetes mellitus and microalbuminuria or overt nephropathy.
Mechanism of action: There are two isoforms of ACE: the somatic isoform, which exists as a glycoprotein comprised of a single polypeptide chain of 1277; and the testicular isoform, which has a lower molecular mass and is thought to play a role in sperm maturation and binding of sperm to the oviduct epithelium. Somatic ACE has two functionally active domains, N and C, which arise from tandem gene duplication. Although the two domains have high sequence similarity, they play distinct physiological roles. The C-domain is predominantly involved in blood pressure regulation while the N-domain plays a role in hematopoietic stem cell differentiation and proliferation. ACE inhibitors bind to and inhibit the activity of both domains, but have much greater affinity for and inhibitory activity against the C-domain. Lisinopril, one of the few ACE inhibitors that is not a prodrug, competes with ATI for binding to ACE and inhibits and enzymatic proteolysis of ATI to ATII. Decreasing ATII levels in the body decreases blood pressure by inhibiting the pressor effects of ATII as described in the Pharmacology section above. Lisinopril also causes an increase in plasma renin activity likely due to a loss of feedback inhibition mediated by ATII on the release of renin and/or stimulation of reflex mechanisms via baroreceptors.
Toxicity: Symptoms of overdose include severe hypotension, electrolyte disturbances, and renal failure. LD50= 2000 mg/kg(orally in rat). Most frequent adverse effects include headache, dizziness, cough, fatigue and diarrhea.
Metabolism: Does not undergo metabolism, excreted unchanged in urine.
Retail: Advanced Pharmaceutical Services Inc., Aidarex Pharmacuticals LLC, Amerisource Health Services Corp., Apotex Inc., Apotheca Inc., A-S Medication Solutions LLC and 69 more suppliers
Lisinopril 10 mg tablet - 1.01 USD / tablet
Prinivil 10 mg tablet - 1.01 USD / tablet
Zestril 2.5 mg tablet - 1.04 USD / tablet
Prinivil 20 mg tablet - 1.05 USD / tablet
Lisinopril 20 mg tablet - 1.09 USD / tablet
Zestoretic 20-12.5 tablet - 1.26 USD / tablet
and 54 more options

(Information from DrugBank)

Compound Target Characteristics

Angiotensin-converting enzyme

Best gene implication in ageing for this target family came from gene M0RB66 via mapping the annotation from Ensembl ENSRNOG00000007467 via mapping the annotation from RGD 2493 annotated in RGD 2014-03-11. Annotation GO 7568 (aging) was Inferred from Expression Pattern

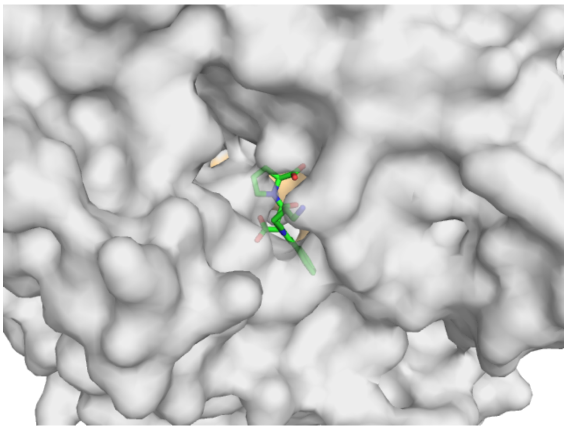

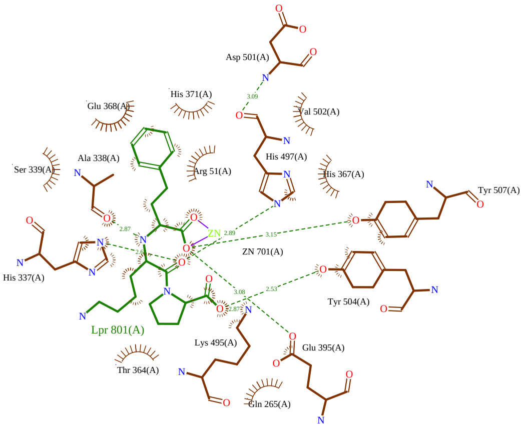

protein	amino acids contacts (binding site)															
PDB:1j36:chainA:Q10714	R	Q	H	A	S	T	H	E	H	E	K	Y	H	V	Y	Y
PDB:2x91:chainA:Q10714	G	Q	H	A	S	T	H	E	H	E	K	Y	H	V	Y	Y
tr:L7MUH0:L7MUH0_HUMAN	N	Q	H	A	S	V	H	E	H	E	K	F	H	V	Y	Y
sp:P12821:ACE_HUMAN	N	Q	H	A	S	V	H	E	H	E	K	F	H	V	Y	Y
tr:M0RB66:M0RB66_RAT	V	Q	H	T	S	S	H	Q	H	E	K	F	H	M	Y	Y
tr:Q8K233:Q8K233_MOUSE	N	Q	H	P	S	I	H	E	H	E	K	F	H	V	Y	Y
sp:P09470:ACE_MOUSE	N	Q	H	P	S	I	H	E	H	E	K	F	H	V	Y	Y
tr:Q3TU20:Q3TU20_MOUSE	N	Q	H	P	S	I	H	E	H	E	K	F	H	V	Y	Y
sp:Q10714:ACE_DROME	G	Q	H	A	S	T	H	E	H	E	K	Y	H	V	Y	Y
sp:Q18581:ACN1_CAEEL	F	G	H	A	A	Q	S	L	Q	D	H	N	S	S	A	T

	whole protein		domain-based		contact-based	
protein	ident	simil	ident	simil	ident	simil
PDB:1j36:chainA:Q10714	0.98	0.99	0.99	1.0	1.0	1.0
PDB:2x91:chainA:Q10714	1.0	1.0	1.0	1.0	0.94	0.93
tr:L7MUH0:L7MUH0_HUMAN	0.35	0.65	0.45	0.8	0.81	0.9
sp:P12821:ACE_HUMAN	0.2	0.37	0.45	0.8	0.81	0.9
tr:M0RB66:M0RB66_RAT	0.31	0.64	0.4	0.79	0.63	0.83
tr:Q8K233:Q8K233_MOUSE	0.25	0.47	0.44	0.8	0.75	0.87
sp:P09470:ACE_MOUSE	0.19	0.36	0.44	0.8	0.75	0.87
tr:Q3TU20:Q3TU20_MOUSE	0.19	0.36	0.44	0.8	0.75	0.87
sp:Q10714:ACE_DROME	1.0	1.0	1.0	1.0	0.94	0.93
sp:Q18581:ACN1_CAEEL	0.16	0.49	0.23	0.71	0.13	0.48

Ance (FBgn0012037) associated phenotypes

partially lethal - majority live

(Information from FlyBase)

Ance (UniProt:Q10714) annotation

Function: May be involved in the specific maturation or degradation of a number of bioactive peptides. May play a role in the contractions of the heart, gut and testes, and in spermatid differentiation. (PubMed:12591244).
Cofactor: Zn(2+)Note=Binds 1 zinc ion per subunit.;
Enzyme regulation: Inhibited by captopril and, to a lesser extent, by lisinopril, trandolaprilat, fosinoprilat and enalaprilat. (PubMed:7775412, PubMed:8761461).
Biophysicochemical properties: Kinetic parameters: KM=33.5 uM for angiotensin I (PubMed:7775412, PubMed:8761461, PubMed:9839949); KM=53.4 uM for N-acetyl-Ser-Asp-Lys-Pro (PubMed:7775412, PubMed:8761461, PubMed:9839949); KM=2.59 mM for Hip-His-Leu (PubMed:7775412, PubMed:8761461, PubMed:9839949); KM=10.26 mM for Hip-His-Leu-NH(2) (PubMed:7775412, PubMed:8761461, PubMed:9839949); KM=372 uM for (Leu5)enkephalin (PubMed:7775412, PubMed:8761461, PubMed:9839949); KM=1.88 mM for (Leu5)enkephalinamide (PubMed:7775412, PubMed:8761461, PubMed:9839949);
Subcellular location: Secreted, extracellular space.

Tissue specificity: Expressed in vesicular structures in spermatocytes and early spermatids (at protein level). (PubMed:12591244).
Developmental stage: Expressed in the amnioserosa during germ band elongation, shortening and heart morphogenesis. Expressed in midgut throughout embryogenesis.
Ptm: Glycosylated. (PubMed:8761461).
Disruption phenotype: Male flies are sterile. (PubMed:12591244).

(Information from UniProt)

acn-1 (WBGene00000039) associated phenotypes
embryonic lethal, lethal, sterile

(Information from WormBase)

acn-1 (UniProt:Q18581) annotation
Function: Required for larval molting, male tail development, and formation of adult alae. Inactive as a metallopeptidase, due to a lack of active site residues. (PubMed:14559923).
Tissue specificity: Expressed in embryonic and larval hypodermis, in the vulva during organogenesis, and in the ray papillae of the male tail. (PubMed:14559923).

(Information from UniProt)
